# Fas–Fas Ligand: Checkpoint of T Cell Functions in Multiple Sclerosis

**DOI:** 10.3389/fimmu.2016.00382

**Published:** 2016-09-27

**Authors:** Elisabetta Volpe, Manolo Sambucci, Luca Battistini, Giovanna Borsellino

**Affiliations:** ^1^Neuroimmunology Unit, Santa Lucia Foundation, Rome, Italy

**Keywords:** Fas–FasL, multiple sclerosis, Thelper 17 cells, cell death, T regulatory cells

## Abstract

Fas and Fas Ligand (FasL) are two molecules involved in the regulation of cell death. Their interaction leads to apoptosis of thymocytes that fail to rearrange correctly their T cell receptor (TCR) genes and of those that recognize self-antigens, a process called negative selection; moreover, Fas–FasL interaction leads to activation-induced cell death, a form of apoptosis induced by repeated TCR stimulation, responsible for the peripheral deletion of activated T cells. Both control mechanisms are particularly relevant in the context of autoimmune diseases, such as multiple sclerosis (MS), where T cells exert an immune response against self-antigens. This concept is well demonstrated by the development of autoimmune diseases in mice and humans with defects in Fas or FasL. In recent years, several new aspects of T cell functions in MS have been elucidated, such as the pathogenic role of T helper (Th) 17 cells and the protective role of T regulatory (Treg) cells. Thus, in this review, we summarize the role of the Fas–FasL pathway, with particular focus on its involvement in MS. We then discuss recent advances concerning the role of Fas–FasL in regulating Th17 and Treg cells’ functions, in the context of MS.

## Introduction

Fas and Fas Ligand (FasL) are members of the tumor necrosis factor (TNF)-receptor and TNF family, respectively. The ligation of Fas with FasL results in the activation of a caspase cascade that initiates apoptosis (1–5).

Apoptosis mediated by Fas–FasL is an important mechanism for the maintenance of immune homeostasis. During a physiological immune response, programed cell death (apoptosis) has the important role to delete potentially pathogenic autoreactive lymphocytes from the circulation and tissues, limiting tissue damage inevitably caused by immune responses (6). In fact, T cell receptor (TCR) restimulation of previously activated and expanded T cells in the absence of appropriate co-stimulation induces activation-induced cell death (AICD) (7–9), an important mechanism for removal of overly activated T cells, such as autoreactive T cells in autoimmune diseases. Multiple sclerosis (MS) is an autoimmune disease characterized by the accumulation of CD4 and CD8 T cells in the central nervous system (CNS) compartment (10, 11). CD8 T cells expand clonally and by targeting specific antigens they are accountable for oligodendrocyte loss, demyelination, and neuronal damage. Although CD4 T cell responses have less substantial clonal features than CD8 T cells, they do expand and accumulate in the brain (10, 12) where they play a critical role in inflammation and in priming CD8 and B cells. The control of the potentially limitless expansion of these cells is achieved also by Fas–FasL-mediated apoptosis, and its therapeutic enhancement could be useful to reduce pathogenic T cells in MS.

## Activation and Regulation of the Fas–FasL Pathway

**Figure 1:**
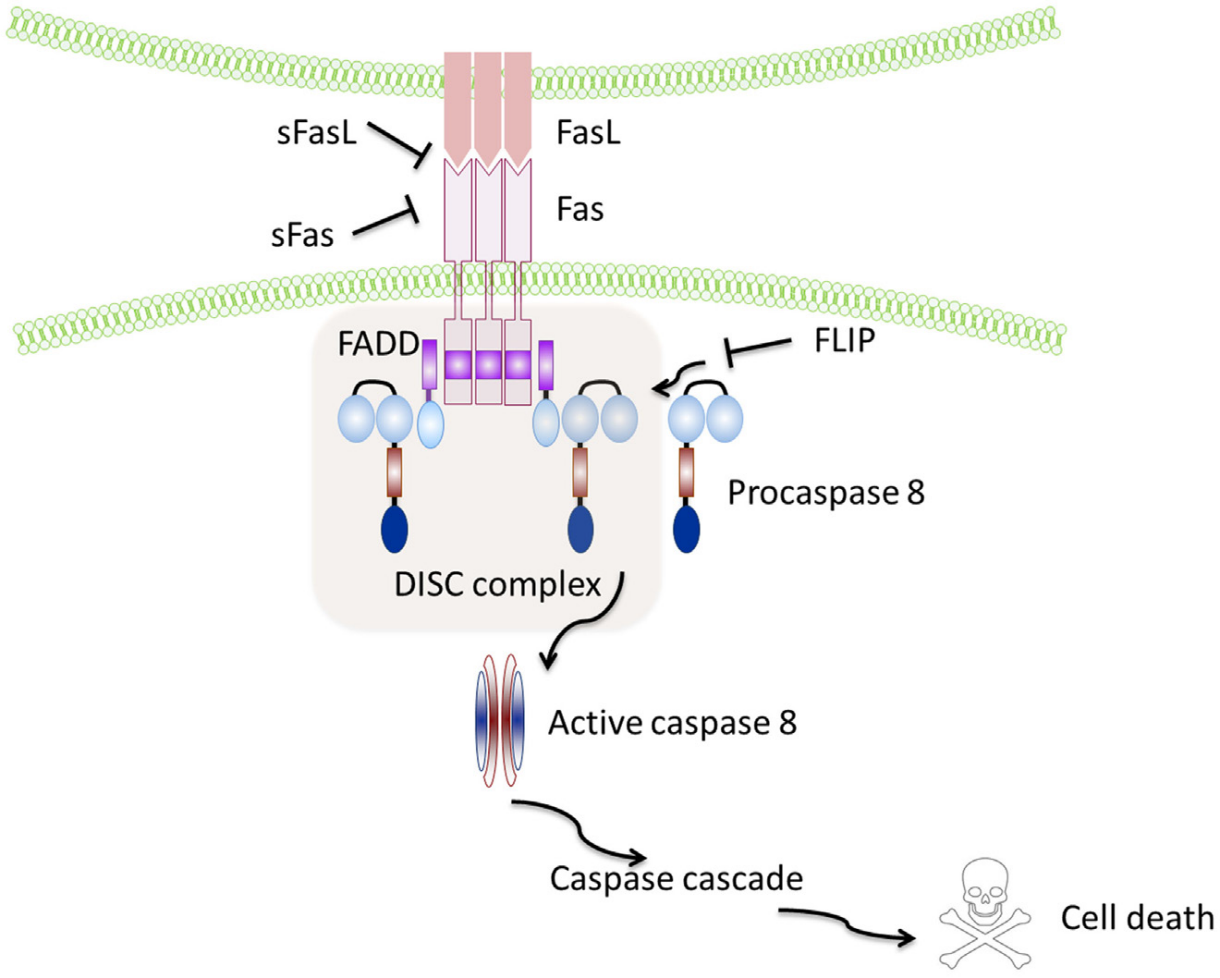
**Schematic representation of the Fas–FasL pathway**. Binding of the Fas leads to recruitment and activation by the protein adaptor FADD of procaspase-8 and formation of the death-inducing signalling complex (DISC). Active caspase-8 directly cleaves caspase-3 and initiates the caspase cascade, which ultimately leads to cell death. Soluble Fas and soluble FasL bind to the respective ligands inhibiting activation of the pathway. FLIP inhibits activation of caspase-8 and is thus a major anti-apoptotic protein.

Fas (also called CD95 or APO-1 or TNFRSF6) is a type I transmembrane protein (2), containing a death domain (DD) in its cytoplasmic region, which is essential for the induction of apoptosis (13). The induction of apoptosis is triggered by the interaction of Fas with its ligand (FasL), a 40-kDa membrane protein (14) allowing recruitment of the adaptor protein Fas-associated death domain (FADD) (15) and binding of procaspase-8, resulting in the formation of the death-inducing signaling complex (DISC) (16, 17), which finally leads to the activation of effector caspase-3 by active Caspase-8 (Figure 1).

The membrane-bound form (mFasL) can be cleaved from the cell surface by metalloproteinases to produce a truncated soluble product (sFasL) of 26 kDa derived from the extracellular domain (14). In the mouse, sFasL can also be generated by alternative splicing (18).

However, it is not clear what triggers sFasL release, but it is plausible that abnormal or excessive activation of T cells causes the production of sFasL, with deleterious systemic effects.

However, sFasL does not activate Fas, and it competes with mFasL reducing its cytotoxic activity (19, 20). This is due to the fact that sFasL binds Fas, but it is unable to induce its oligomerization, thus preventing activation of the proapoptotic signaling pathway by mFasL (21–23) (Figure 1).

Similar to FasL, a membrane and soluble isoform with opposite functions have also been described for Fas: the isoform skipped of exon 6, that encodes the transmembrane region, leads to the synthesis of an mRNA that codes for a soluble form of the receptor known to repress apoptosis (24, 25), and the Ewing sarcoma protein (EWS), which has been recently described as responsible for the Fas splicing event (26). Notably, the anti-apoptotic protein caspase-8 (FLICE)-like inhibitory protein (FLIP) is another potent inhibitor of Fas signaling that may block Fas-mediated apoptosis by disturbing the formation of the DISC (27) (Figure 1).

The activation of the Fas–FasL pathway is finely regulated by several mechanisms, including formation of Fas microclusters (21, 28–31), actin reorganization (31), inducible or constitutive association with membrane rafts (32–36), and acid sphingomyelinase-mediated ceramide production (37).

Moreover, another important mechanism of regulation of cell death mediated by Fas–FasL is the transcriptional control of FasL gene expression. Indeed, while Fas is ubiquitously expressed in a variety of tissues and with particular abundance in the thymus, liver, and kidney (38), FasL expression is controlled by specific protein-DNA interactions at the FasL promoter. Several factors have been identified, which regulate FasL gene expression, such as specificity protein-1 (Sp1); Ets-1 (the homolog of viral Ets); interferon regulatory factor (IRF) 1 and 2; inducible cAMP early repressor (ICER); nuclear factor in activated T cells (NFAT); nuclear factor-kappa B (NF-kB); activator protein-1 (AP-1); early growth factor (EGR) 1, 2, and 3; and c-Myc (KAVURMA). Moreover, the modulation of these transcription factors is strictly dependent on environmental cues, including cytotoxic stress, DNA-damaging agents, and interleukin (IL)-2, which promote FasL expression, IL-6, transforming growth factor-beta (TGF-β), retinoic acid, nitric oxide, and Vitamin D3 that repress FasL expression (39).

## The Fas–FasL Activation Pathway in Immune Responses

**Figure 2:**
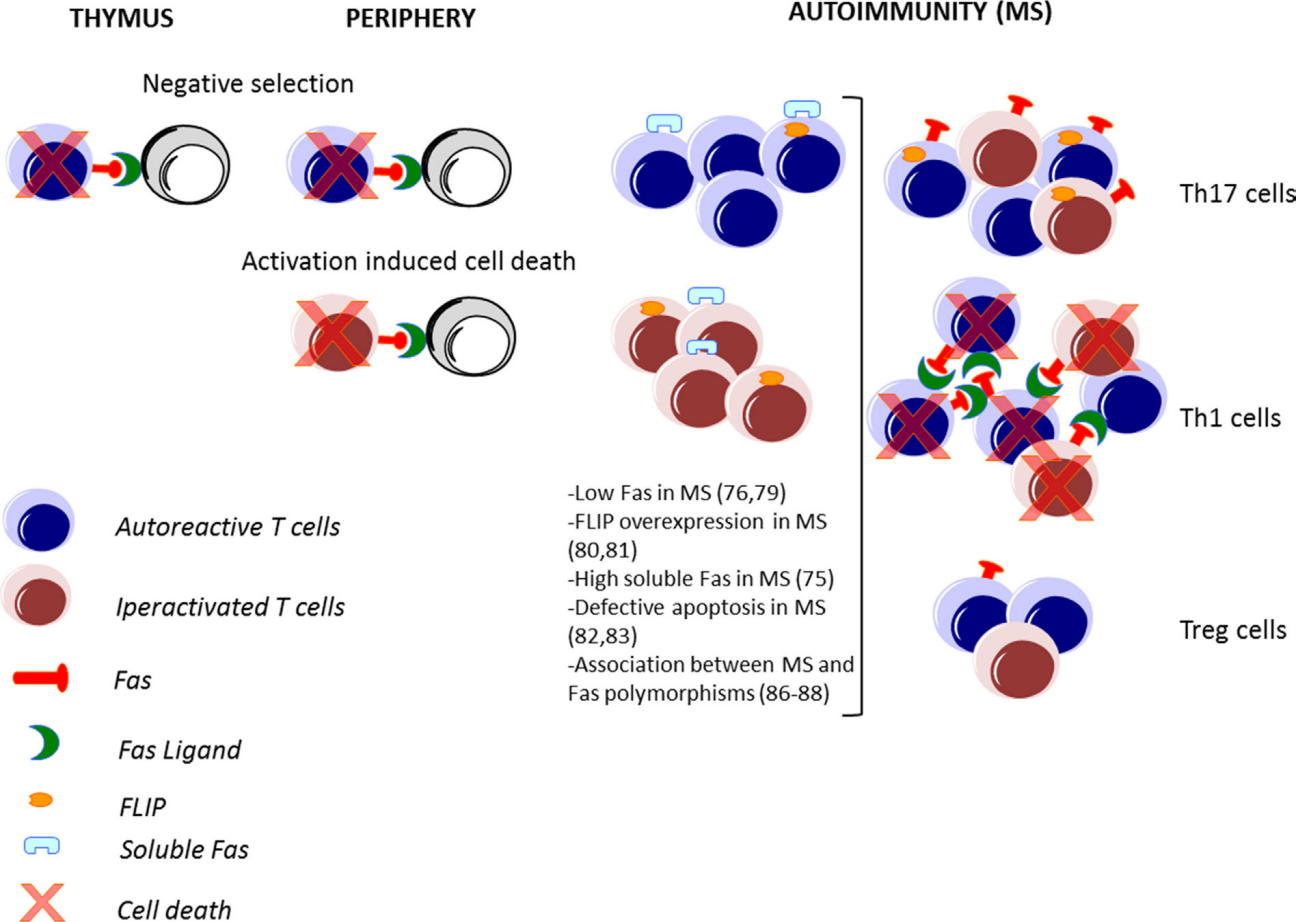
**Fas–FasL-mediated T cell death in immune response and in MS**. The Fas–FasL pathways are not only involved in deletion of autoreactive lymphocytes during the immune system’s development in the thymus and in the periphery (negative selection) but also in the deletion of lymphocytes activated during an immune response (activation-induced cell death). In autoimmune disorders, such as multiple sclerosis (MS), alteration of these processes may lead to a defective deletion and an accumulation of autoreactive and activated T cells. Pathogenic T helper (Th) 1 and Th17 cells are abundant in MS, while protective T regulatory (Treg) cells are less frequent. Moreover, high expression of FasL in Th1, high expression of FLIP by Th17 cells, and low expression of Fas by Tregs lead to a differential cell death sensitivity by Th1, Th17, and Treg cells.

The Fas–FasL-mediated death plays a major role in immune homeostasis: it is required for the deletion of autoreactive lymphocytes during the immune system’s development (negative selection); this process is defined as central tolerance in the thymus (40) and peripheral tolerance in the periphery (41), and it is also required for the control of the number of lymphocytes activated during an immune response against a pathogen, leading to the contraction of the ongoing immune response (42) (Figure 2).

Moreover, the Fas–FasL pathway is required not only for death of T cells (43) but also for deletion of autoreactive B cells (44, 45), B cell somatic hypermutation (46), cytotoxicity of NK and CD8 T cells (47, 48), apoptosis of endothelial cells (49), regulation of myeloid suppressor cells’ turnover (50), and activation of macrophages’ functions against infections (51).

The Fas–FasL interaction was also described as an important mechanism leading to immune privilege in specialized tissues, such as the CNS, eye, testis, ovary, pregnant uterus, and placenta, through the induction of apoptosis in infiltrating inflammatory cells (52–54).

However, the CNS is no longer considered an immune privileged site in a strict sense and indeed immune cells, especially T cells can be detected in the CNS under normal conditions (55, 56). In this view, the only element protecting the CNS from immune-mediated attacks is the presence of an intact blood–brain barrier (BBB) and the absence of an immune-competent population of tissue macrophages/tissue dendritic cells. Therefore, the expression of FasL by microvascular endothelial cells and astrocytic foot processes, major constituents of the BBB, favors an immune-suppressive environment within the CNS (57).

## The Fas–FasL Activation Pathway in Diseases

Given the crucial role of the Fas–FasL pathway in regulating the balance between cell survival and cell death, it is also implicated in the protection from transplant rejection, tumors, and autoimmunity. The discovery that mice defective in Fas or FasL develop a lymphoproliferation phenotype (lpr) or a generalized-lymphoproliferative-disease phenotype (gld) (1, 5, 58), definitely associated the Fas–FasL pathway to pathology. In humans, patients with mutations in the DD of Fas (Canale–Smith syndrome or autoimmune lymphoproliferative syndrome) have increased numbers of circulating double-negative T cells (>20%), lymphadenopathy, and signs of autoimmunity, such as hemolytic anemia, thrombocytopenia (59), and expansion of autoantibody-secreting plasma cells (46).

However, given the multiple roles of Fas–FasL in immune responses and diseases, therapeutic targeting of the Fas/FasL pathway might not only fail to protect against a specific disease but could also potentially affect the behavior of different cell targets thus influencing the outcome for the patient.

For instance, in tumors, activation of Fas has antagonistic effects: it can lead to tumor apoptosis (60–62) or to tumor cell survival (63, 64). Indeed, although Fas activation can lead to the killing of tumor cells, it can also lead to apoptosis of infiltrating lymphocytes. In this context, there are several evidences that show that the constitutive expression of FasL by tumor cells can be used as a mechanism of immune evasion (65) by directly leading to apoptosis of infiltrating Fas positive lymphocytes (66, 67).

It is becoming clear that a potential therapy targeting the Fas–FasL pathway in disease should consider the direct targeting of the pathogenic cells for specific diseases, such as tumor cells for cancer and immune cells for autoimmunity.

## The Fas–FasL Pathway in Multiple Sclerosis

Multiple sclerosis is an autoimmune disease of the CNS characterized by demyelination and axon damage caused by infiltration of inflammatory cells, including autoreactive lymphocytes and macrophages (68). The first evidence for a role of Fas–FasL system in MS stems from the observation that Fas and FasL are expressed in brain lesions of MS patients. In particular, FasL is expressed by astrocytes, oligodendrocytes, and macrophages, while Fas is mainly expressed by macrophages, T cells, and oligodendrocytes (69, 70).

Several studies have addressed the role of the Fas–FasL system in experimental autoimmune encephalomyelitis (EAE), the murine model of MS (71–75). Mice carrying mutations in Fas (lpr) or FasL (gld) generally show a milder disease course, despite persistence of immune cell infiltrates into the CNS. Fas expression by neural cells, particularly oligodendrocytes, seems to be important for disease progression, and lpr mice show fewer cells undergoing apoptosis in the CNS (74); on the other hand, lack of FasL on pathogenic lymphocytes transferred for the induction of EAE determines attenuated (71, 75) and monophasic (72) disease. Moreover, FasL-deficient mice develop prolonged signs of EAE when immunized with wild-type autoreactive T cells, indicating that in autoreactive T cells, the Fas–FasL system plays a regulatory role during the recovery from EAE. Thus, the Fas–FasL pathway is involved in the development and/or progression of autoimmunity in the CNS.

Further studies investigating the role of the Fas–FasL system in MS have been performed in human samples through the analysis of peripheral blood cells, serum, and cerebrospinal fluid (CSF) from MS patients. Serum levels of soluble Fas are significantly elevated in patients with relapsing remitting MS (76), indicating that it could contribute to inhibit apoptosis in this disease. Consistent with these results, it has been reported that Fas transcript is decreased in the active phase of MS patients (77), and Fas expression on the surface of CD4^+^ CCR5^+^ T cells, a T cell subset implicated in MS (78, 79), is decreased in MS patients compared to healthy donors (HD) (80). Moreover, the inhibitor protein FLIP is overexpressed in intrathecal and blood lymphocytes from MS patients (81, 82). These results suggest that the Fas–FasL pathway is affected in MS, and this phenomenon could lead to defective cell death and thus to increased survival of pathogenic cells. This hypothesis was confirmed by functional studies that revealed a defective sensitivity to apoptosis of blood T cells derived from MS patients compared to cells from HD (83, 84).

In contrast, it is not clear whether FasL expression is increased or decreased in activated peripheral blood mononuclear cells from MS patients compared to HD, as reported by two studies describing conflicting results (85, 86). However, studies on peripheral blood mononuclear cells should focus on distinct cell subsets rather than on the bulk population of lymphocytes, particularly when the cells of interest are present at low frequency. Failing to do so may explain the reported discordant results on levels of FasL expression, since comparative studies between individuals may be affected by differential representation of functionally distinct subsets.

Differential expression of Fas and FasL could also be a result of a differential genetic regulation in MS and HD. However, genetic studies consistently demonstrate only a weak association between MS and Fas polymorphisms (87–89).

## Fas–FasL Pathway in T Cells with Pathogenic Role in MS

The T cell population includes a variety of T cell subsets. In recent years, two subsets emerged as particularly relevant in MS disease: T helper (Th) 17 that produce IL-17 (90–92) and T regulatory (Treg) cells that suppress the functions of effector T cells (93).

In particular, the increased expression of IL-17 produced by Th17 cells has been associated with MS (94), and its inhibition or deletion in the corresponding animal model has provided varying degrees of protection (95). In contrast, Treg cells are present at lower frequency in patients with MS and are defective in their suppressor functions *in vitro* (96, 97). Indeed, there is a functional antagonism between Th17 and Treg cells, and the increase of Th17 cells and a decrease of Treg cells observed in MS patients compared to HD indicate an important role of the Th17/Treg balance in the modulation of MS disease. Thus, the impact of the Fas–FasL system could differentially regulate MS disease, depending on the T cell target (Figure 2).

Several studies have demonstrated that murine Th17 cells are more resistant to AICD than another Th subset called Th1, characterized by predominant and abundant interferon (IFN)-γ production (98–100). Th1 cells have a pathogenic role in MS (101), particularly in the initiation of the inflammatory response, through the activation of macrophages (102) and the induction of increased vascular adherence that facilitates access in the CNS of the critical effector cells sustaining tissue damage, such as Th17 cells (103).

Interestingly, differential cell death sensitivity between Th1 and Th17 cells is also confirmed in cells derived from MS patients (100). Since the homeostatic regulation of cell expansion by cell death is similar in HD and MS patients, the persistence of Th17 cells in MS disease may be due to altered mechanisms of Th17 cell generation in MS patients compared to HD. Thus, this process could be responsible for the impaired apoptotic deletion of polyclonal and myelin-specific T cells derived from MS patients’ blood (83). In fact, the impaired apoptotic deletion observed in MS could be related to the higher frequency of apoptosis-resistant cell subsets in MS compared to HD (104).

Similar to Th17 cells, Th1/17 (coproducing IL-17 and IFN-γ) cells resist to AICD, suggesting that this mechanism could also be responsible for the persistence of cells producing both IL-17 and IFN-γ, emerging as potentially relevant in the pathogenesis of MS (105).

Interestingly, low FasL and FLIP expression in Th17 cells compared to Th1 cells are the major mechanisms regulating their differential cell death sensitivity (98–100) (Figure 2). Recently, it has been demonstrated that low levels of mitogen-activated protein kinases (MAPKs), such as Erk1/2 and p38α, upon TCR stimulation, alter FasL expression and AICD sensitivity of Th17 cells (106).

In MS, the involvement of FasL has been largely investigated in several studies as mentioned above, but contrasting results have been reported (85, 86). Thus, the differences in Th subset representation reported in those studies may explain the discordant results on the level of FasL expression in total lymphocytes from HD and MS patients. The lack of expression of FasL by Th17 and Th1/17 cells suggests that where generation of IL-17-producing cells is favored or increased, as in MS, accumulation of FasL negative cells in inflammatory sites may preclude interactions with FasL expressing cells, determining an escape from homeostatic containment.

Another important source of IL-17 in MS is the CD161^+^ CD8^+^ T cell population, called mucosal-associated invariant T (MAIT) cells, which have been recently identified also within MS lesions (107, 108). There are evidences showing that these cells resist to cell death induced by chemotherapy due to the high levels of the multidrug receptor ABCB1 (also called P-gp, MDR1, and PGY1), which can rapidly efflux xenobiotics (109). MAIT cells express high levels of Fas (108), indicating their potential susceptibility to Fas-mediated cell death. However, investigations on the functionality of Fas–FasL pathways in these cells need to be performed.

## Fas–FasL Pathway in T Cells with Protective Role in MS

Fas–FasL is also involved in the regulation of cells known to have a protective role in MS, such as Treg cells (96, 97). In particular, apoptosis mediates homeostasis of Treg cells and Treg cell-mediated suppression (110). Treg cells with a CD4^+^ CD25^high^ Forkhead box P3 (Foxp3)^+^ phenotype include a distinct subset of lymphocytes programed in the thymus (called naturally occurring Tregs) and adaptive Treg cells generated from naive CD4 T cells in the periphery (111).

The study of the expression of surface molecules involved in apoptosis revealed that FasL is expressed at low levels in human and murine Treg cells upon stimulation (112), and that Fas is present at particularly low levels in a small subset of Tregs expressing CD45RA, a hallmark of resting/naive T cells (113, 114), which are thus resistant to apoptosis induced by exogenous Fas stimulation (115) (Figure 2). However, these cells are sensitive to FasL-induced apoptosis in the absence of TCR stimulation (116). In the site of inflammation, the sensitivity to apoptosis of Treg cells is modulated by several factors, including proliferation, cytokine environment, and antigenic stimulation (110, 117). For instance, activation-associated proliferation renders T effector cells more susceptible to AICD than Treg cells; IL-2 promotes AICD through the induction of FasL expression in activated T effector cells but does not sensitize Treg cells to AICD (117); TGF-β produced by Treg cells protects them from apoptotic death (118); and TCR engagement and CD3 cross-linking induce cell death in T effector cells and not in Treg cells (116).

The different expression patterns of Fas and FasL of Treg cells compared to conventional effector lymphocytes might be due to regulation by Foxp3, the master transcription factor of Treg cells, which negatively regulates AICD and FasL expression. Indeed, in human Treg cells, knockdown of Foxp3 partially rescues FasL expression and AICD, and in mouse, Foxp3-mutant Treg cells from Scurfy mice express FasL at levels similar to those of conventional T cells (112). Interestingly, a genome-wide screen for Foxp3 target genes revealed a binding of Foxp3 in proximity to the FasL gene, but its direct interaction remains to be determined (119, 120).

The Fas–FasL pathway in Treg cells obtained from MS patients has never been investigated; however, in another human chronic inflammatory disease, acute coronary syndrome, an alteration in the Fas/FasL pathway in Treg cells was described: here, in fact, Treg but not Th17 cells are sensitive to Fas-mediated apoptosis, and this could determine an imbalance between these two subsets, favoring inflammation (120). It was also shown that Treg cells infiltrating the inflamed liver express high levels of Fas and are particularly susceptible to apoptosis, consistent with the observed Treg dysfunction in inflamed tissues. Further studies are expected to shed light on the susceptibility of Treg cells to apoptosis in distinct disease models, to better understand how the experimental conditions affect their susceptibility to apoptosis, and to establish potential differences between thymic and adaptative Treg cells.

## Conclusion

The Fas–FasL pathway regulates cell death of several cell types, and given the broad expression of this pathway, it is important to define the specific role of each cell type in specific diseases. In particular, Fas–FasL regulates T cell functions and certainly contributes to diseases where T cells play a major role, as MS. However, several T cell subsets have been described, and in MS, they may have antagonistic roles: Th17 play a pathogenic role, while Treg cells exert a protective role by suppressing pathogenic effector T cells. The low FasL expression in Th17 cells indicates that these cells have evolved a mechanism to escape the programed cell death and to persist in inflamed sites. FasL is expressed at low levels also by Treg cells, possibly to enable their prolonged survival necessary to dampen immune reactions once the inflammatory stimulus has subsided.

Consequently, an intriguing challenge for reducing inflammatory responses in MS would be to find a strategy to simultaneously induce specific cell killing of Th17 cells and to potentiate cell survival of protective Treg cells.

## Author Contributions

EV organized the different sections of the manuscript and wrote the manuscript; MS wrote the section on T regulatory cells; LB critically reviewed the manuscript for important intellectual content; and GB coordinated author contributions and finalized the manuscript for submission. All authors approved the final version.

## Conflict of Interest Statement

The authors declare that the research was conducted in the absence of any commercial or financial relationships that could be construed as a potential conflict of interest.

## References

[1] Nagata S Golstein P. The Fas death factor. Science (1995) 267(5203):1449–56. 10.1126/science.7533326 7533326

[2] Ashkenazi A Dixit VM. Death receptors: signaling and modulation. Science (1998) 281(5381):1305–8. 10.1126/science.281.5381.1305 9721089

[3] Ashkenazi A Dixit VM. Apoptosis control by death and decoy receptors. Curr Opin Cell Biol (1999) 11(2):255–60. 10.1016/S0955-0674(99)80034-9 10209153

[4] Wolf BB Green DR Suicidal tendencies: apoptotic cell death by caspase family proteinases. J Biol Chem (1999) 274(29):20049–52. 10.1074/jbc.274.29.20049 10400609

[5] Nagata S Apoptosis by death factor. Cell (1997) 88(3):355–65. 10.1016/S0092-8674(00)81874-7 9039262

[6] Chervonsky AV. Apoptotic and effector pathways in autoimmunity. Curr Opin Immunol (1999) 11(6):684–8. 10.1016/S0952-7915(99)00037-0 10631555

[7] Mercep M Weissman AM Frank SJ Klausner RD Ashwell JD. Activation-driven programmed cell death and T cell receptor zeta eta expression. Science (1989) 246(4934):1162–5. 10.1126/science.2531464 2531464

[8] Shi YF Sahai BM Green DR. Cyclosporin A inhibits activation-induced cell death in T-cell hybridomas and thymocytes. Nature (1989) 339(6226):625–6. 10.1038/339625a0 2786609

[9] Krammer PH. CD95’s deadly mission in the immune system. Nature (2000) 407(6805):789–95. 10.1038/35037728 11048730

[10] Hemmer B Archelos JJ Hartung HP New concepts in the immunopathogenesis of multiple sclerosis. Nat Rev Neurosci (2002) 3(4):291–301. 10.1038/nrn784 11967559

[11] Dendrou CA Fugger L Friese MA. Immunopathology of multiple sclerosis. Nat Rev Immunol (2015) 15(9):545–58. 10.1038/nri3871 26250739

[12] Foulds KE Zenewicz LA Shedlock DJ Jiang J Troy AE Shen H. Cutting edge: CD4 and CD8 T cells are intrinsically different in their proliferative responses. J Immunol (2002) 168(4):1528–32. 10.4049/jimmunol.168.4.1528 11823476

[13] Fesik SW Insights into programmed cell death through structural biology. Cell (2000) 103(2):273–82. 10.1016/S0092-8674(00)00119-7 11057900

[14] Tanaka M Suda T Takahashi T Nagata S. Expression of the functional soluble form of human fas ligand in activated lymphocytes. EMBO J (1995) 14(6):1129–35. 7536672 10.1002/j.1460-2075.1995.tb07096.x PMC398190

[15] Peter ME Krammer PH. The CD95(APO-1/Fas) DISC and beyond. Cell Death Differ (2003) 10(1):26–35. 10.1038/sj.cdd.4401186 12655293

[16] Boatright KM Renatus M Scott FL Sperandio S Shin H Pedersen IM A unified model for apical caspase activation. Mol Cell (2003) 11(2):529–41. 10.1016/S1097-2765(03)00051-0 12620239

[17] Donepudi M Mac Sweeney A Briand C Grutter MG. Insights into the regulatory mechanism for caspase-8 activation. Mol Cell (2003) 11(2):543–9. 10.1016/S1097-2765(03)00059-5 12620240

[18] Ayroldi E D’Adamio F Zollo O Agostini M Moraca R Cannarile L Cloning and expression of a short Fas ligand: a new alternatively spliced product of the mouse Fas ligand gene. Blood (1999) 94(10):3456–67. 10552956

[19] Suda T Hashimoto H Tanaka M Ochi T Nagata S. Membrane Fas ligand kills human peripheral blood T lymphocytes, and soluble Fas ligand blocks the killing. J Exp Med (1997) 186(12):2045–50. 10.1084/jem.186.12.2045 9396774 PMC2199173

[20] Schneider P Holler N Bodmer JL Hahne M Frei K Fontana A Conversion of membrane-bound Fas(CD95) ligand to its soluble form is associated with downregulation of its proapoptotic activity and loss of liver toxicity. J Exp Med (1998) 187(8):1205–13. 10.1084/jem.187.8.1205 9547332 PMC2212219

[21] Varadhachary AS Edidin M Hanlon AM Peter ME Krammer PH Salgame P. Phosphatidylinositol 3’-kinase blocks CD95 aggregation and caspase-8 cleavage at the death-inducing signaling complex by modulating lateral diffusion of CD95. J Immunol (2001) 166(11):6564–9. 10.4049/jimmunol.166.11.6564 11359808

[22] Huang DC Hahne M Schroeter M Frei K Fontana A Villunger A Activation of Fas by FasL induces apoptosis by a mechanism that cannot be blocked by Bcl-2 or Bcl-x(L). Proc Natl Acad Sci U S A (1999) 96(26):14871–6. 10.1073/pnas.96.26.14871 10611305 PMC24740

[23] Jang S Krammer PH Salgame P. Lack of proapoptotic activity of soluble CD95 ligand is due to its failure to induce CD95 oligomers. J Interferon Cytokine Res (2003) 23(8):441–7. 10.1089/107999003322277856 13678432

[24] Cheng J Zhou T Liu C Shapiro JP Brauer MJ Kiefer MC Protection from Fas-mediated apoptosis by a soluble form of the Fas molecule. Science (1994) 263(5154):1759–62. 10.1126/science.7510905 7510905

[25] Cascino I Fiucci G Papoff G Ruberti G. Three functional soluble forms of the human apoptosis-inducing Fas molecule are produced by alternative splicing. J Immunol (1995) 154(6):2706–13. 7533181

[26] Paronetto MP Bernardis I Volpe E Bechara E Sebestyen E Eyras E Regulation of FAS exon definition and apoptosis by the Ewing sarcoma protein. Cell Rep (2014) 7(4):1211–26. 10.1016/j.celrep.2014.03.077 24813895

[27] Irmler M Thome M Hahne M Schneider P Hofmann K Steiner V Inhibition of death receptor signals by cellular FLIP. Nature (1997) 388(6638):190–5. 10.1038/40657 9217161

[28] Kischkel FC Hellbardt S Behrmann I Germer M Pawlita M Krammer PH Cytotoxicity-dependent APO-1 (Fas/CD95)-associated proteins form a death-inducing signaling complex (DISC) with the receptor. EMBO J (1995) 14(22):5579–88. 8521815 10.1002/j.1460-2075.1995.tb00245.x PMC394672

[29] Kamitani T Nguyen HP Yeh ET. Activation-induced aggregation and processing of the human Fas antigen. Detection with cytoplasmic domain-specific antibodies. J Biol Chem (1997) 272(35):22307–14. 10.1074/jbc.272.35.22307 9268381

[30] Ruiz-Ruiz C Robledo G Font J Izquierdo M Lopez-Rivas A. Protein kinase C inhibits CD95 (Fas/APO-1)-mediated apoptosis by at least two different mechanisms in Jurkat T cells. J Immunol (1999) 163(9):4737–46. 10528172

[31] Algeciras-Schimnich A Shen L Barnhart BC Murmann AE Burkhardt JK Peter ME. Molecular ordering of the initial signaling events of CD95. Mol Cell Biol (2002) 22(1):207–20. 10.1128/MCB.22.1.207-220.2002 11739735 PMC134211

[32] Grassme H Jekle A Riehle A Schwarz H Berger J Sandhoff K CD95 signaling via ceramide-rich membrane rafts. J Biol Chem (2001) 276(23):20589–96. 10.1074/jbc.M101207200 11279185

[33] Grassme H Schwarz H Gulbins E. Molecular mechanisms of ceramide-mediated CD95 clustering. Biochem Biophys Res Commun (2001) 284(4):1016–30. 10.1006/bbrc.2001.5045 11409897

[34] Hueber A Welsandt G Jordan JF Mietz H Weller M Krieglstein GK Characterization of CD95 ligand (CD95L)-induced apoptosis in human tenon fibroblasts. Exp Eye Res (2002) 75(1):1–8. 10.1006/exer.2001.1171 12123632

[35] Aouad SM Cohen LY Sharif-Askari E Haddad EK Alam A Sekaly RP. Caspase-3 is a component of Fas death-inducing signaling complex in lipid rafts and its activity is required for complete caspase-8 activation during Fas-mediated cell death. J Immunol (2004) 172(4):2316–23. 10.4049/jimmunol.172.4.2316 14764700

[36] Muppidi JR Siegel RM. Ligand-independent redistribution of Fas (CD95) into lipid rafts mediates clonotypic T cell death. Nat Immunol (2004) 5(2):182–9. 10.1038/ni1024 14745445

[37] Cremesti A Paris F Grassme H Holler N Tschopp J Fuks Z Ceramide enables fas to cap and kill. J Biol Chem (2001) 276(26):23954–61. 10.1074/jbc.M101866200 11287428

[38] Klas C Debatin KM Jonker RR Krammer PH. Activation interferes with the APO-1 pathway in mature human T cells. Int Immunol (1993) 5(6):625–30. 10.1093/intimm/5.6.625 7688561

[39] Kavurma MM Khachigian LM. Signaling and transcriptional control of Fas ligand gene expression. Cell Death Differ (2003) 10(1):36–44. 10.1038/sj.cdd.4401179 12655294

[40] Castro JE Listman JA Jacobson BA Wang Y Lopez PA Ju S Fas modulation of apoptosis during negative selection of thymocytes. Immunity (1996) 5(6):617–27. 10.1016/S1074-7613(00)80275-7 8986720

[41] Bouillet P O’Reilly LA. CD95, BIM and T cell homeostasis. Nat Rev Immunol (2009) 9(7):514–9. 10.1038/nri2570 19543226

[42] Green DR Droin N Pinkoski M Activation-induced cell death in T cells. Immunol Rev (2003) 193:70–81. 10.1034/j.1600-065X.2003.00051.x 12752672

[43] Russell JH Rush B Weaver C Wang R. Mature T cells of autoimmune lpr/lpr mice have a defect in antigen-stimulated suicide. Proc Natl Acad Sci U S A (1993) 90(10):4409–13. 10.1073/pnas.90.10.4409 8506280 PMC46520

[44] Rathmell JC Cooke MP Ho WY Grein J Townsend SE Davis MM CD95 (Fas)-dependent elimination of self-reactive B cells upon interaction with CD4+ T cells. Nature (1995) 376(6536):181–4. 10.1038/376181a0 7603571

[45] Fukuyama H Adachi M Suematsu S Miwa K Suda T Yoshida N Requirement of Fas expression in B cells for tolerance induction. Eur J Immunol (2002) 32(1):223–30. 10.1002/1521-4141(200201)32:1<223::AID-IMMU223>3.0.CO;2-4 11782013

[46] Butt D Chan TD Bourne K Hermes JR Nguyen A Statham A FAS inactivation releases unconventional germinal center B cells that escape antigen control and drive IgE and autoantibody production. Immunity (2015) 42(5):890–902. 10.1016/j.immuni.2015.04.010 25979420

[47] Rouvier E Luciani MF Golstein P Fas involvement in Ca(2+)-independent T cell-mediated cytotoxicity. J Exp Med (1993) 177(1):195–200. 10.1084/jem.177.1.195 7678113 PMC2190860

[48] Ramsdell F Seaman MS Miller RE Tough TW Alderson MR Lynch DH. gld/gld mice are unable to express a functional ligand for Fas. Eur J Immunol (1994) 24(4):928–33. 10.1002/eji.1830240422 7512035

[49] Janin A Deschaumes C Daneshpouy M Estaquier J Micic-Polianski J Rajagopalan-Levasseur P CD95 engagement induces disseminated endothelial cell apoptosis in vivo: immunopathologic implications. Blood (2002) 99(8):2940–7. 10.1182/blood.V99.8.2940 11929785

[50] Sinha P Chornoguz O Clements VK Artemenko KA Zubarev RA Ostrand-Rosenberg S. Myeloid-derived suppressor cells express the death receptor Fas and apoptose in response to T cell-expressed FasL. Blood (2011) 117(20):5381–90. 10.1182/blood-2010-11-321752 21450901 PMC3109712

[51] Chakour R Allenbach C Desgranges F Charmoy M Mauel J Garcia I A new function of the Fas-FasL pathway in macrophage activation. J Leukoc Biol (2009) 86(1):81–90. 10.1189/jlb.1008590 19380712

[52] Griffith TS Brunner T Fletcher SM Green DR Ferguson TA. Fas ligand-induced apoptosis as a mechanism of immune privilege. Science (1995) 270(5239):1189–92. 10.1126/science.270.5239.1189 7502042

[53] Choi C Benveniste EN. Fas ligand/Fas system in the brain: regulator of immune and apoptotic responses. Brain Res Brain Res Rev (2004) 44(1):65–81. 10.1016/j.brainresrev.2003.08.007 14739003

[54] Griffith TS Ferguson TA The role of FasL-induced apoptosis in immune privilege. Immunol Today (1997) 18(5):240–4. 10.1016/S0167-5699(97)81663-5 9153956

[55] Flugel A Bradl M. New tools to trace populations of inflammatory cells in the CNS. Glia (2001) 36(2):125–36. 10.1002/glia.1102 11596121

[56] Flugel A Berkowicz T Ritter T Labeur M Jenne DE Li Z Migratory activity and functional changes of green fluorescent effector cells before and during experimental autoimmune encephalomyelitis. Immunity (2001) 14(5):547–60. 10.1016/S1074-7613(01)00143-1 11371357

[57] Carson MJ Doose JM Melchior B Schmid CD Ploix CC. CNS immune privilege: hiding in plain sight. Immunol Rev (2006) 213:48–65. 10.1111/j.1600-065X.2006.00441.x 16972896 PMC2633103

[58] Nagata S Suda T. Fas and Fas ligand: lpr and gld mutations. Immunol Today (1995) 16(1):39–43. 10.1016/0167-5699(95)80069-7 7533498

[59] Drappa J Vaishnaw AK Sullivan KE Chu JL Elkon KB. Fas gene mutations in the Canale-Smith syndrome, an inherited lymphoproliferative disorder associated with autoimmunity. N Engl J Med (1996) 335(22):1643–9. 10.1056/NEJM199611283352204 8929361

[60] Hohlbaum AM Saff RR Marshak-Rothstein A Fas-ligand – iron fist or Achilles’ heel? Clin Immunol (2002) 103(1):1–6. 10.1006/clim.2001.5165 11987979

[61] Krammer PH Galle PR Moller P Debatin KM CD95(APO-1/Fas)-mediated apoptosis in normal and malignant liver, colon, and hematopoietic cells. Adv Cancer Res (1998) 75:251–73. 10.1016/S0065-230X(08)60744-7 9709812

[62] Modiano JF Sun J Lang J Vacano G Patterson D Chan D Fas ligand-dependent suppression of autoimmunity via recruitment and subsequent termination of activated T cells. Clin Immunol (2004) 112(1):54–65. 10.1016/j.clim.2004.03.011 15207782

[63] Hadji A Ceppi P Murmann AE Brockway S Pattanayak A Bhinder B Death induced by CD95 or CD95 ligand elimination. Cell Rep (2014) 7(1):208–22. 10.1016/j.celrep.2014.02.035 24656822 PMC4083055

[64] Peter ME Hadji A Murmann AE Brockway S Putzbach W Pattanayak A The role of CD95 and CD95 ligand in cancer. Cell Death Differ (2015) 22(5):885–6. 10.1038/cdd.2015.25 25849030 PMC4392084

[65] Chappell DB Restifo NP. T cell-tumor cell: a fatal interaction? Cancer Immunol Immunother (1998) 47(2):65–71. 10.1007/s002620050505 9769114 PMC2249694

[66] Hahne M Rimoldi D Schroter M Romero P Schreier M French LE Melanoma cell expression of Fas(Apo-1/CD95) ligand: implications for tumor immune escape. Science (1996) 274(5291):1363–6. 10.1126/science.274.5291.1363 8910274

[67] Bennett MW O’Connell J O’Sullivan GC Brady C Roche D Collins JK The Fas counterattack in vivo: apoptotic depletion of tumor-infiltrating lymphocytes associated with Fas ligand expression by human esophageal carcinoma. J Immunol (1998) 160(11):5669–75. 9605174

[68] Sospedra M Martin R. Immunology of multiple sclerosis. Annu Rev Immunol (2005) 23:683–747. 10.1146/annurev.immunol.23.021704.115707 15771584

[69] Dowling P Husar W Menonna J Donnenfeld H Cook S Sidhu M. Cell death and birth in multiple sclerosis brain. J Neurol Sci (1997) 149(1):1–11. 10.1016/S0022-510X(97)05213-1 9168159

[70] D’Souza SD Bonetti B Balasingam V Cashman NR Barker PA Troutt AB Multiple sclerosis: Fas signaling in oligodendrocyte cell death. J Exp Med (1996) 184(6):2361–70. 10.1084/jem.184.6.2361 8976190 PMC2196365

[71] Dittel BN Merchant RM Janeway CA Jr. Evidence for Fas-dependent and Fas-independent mechanisms in the pathogenesis of experimental autoimmune encephalomyelitis. J Immunol (1999) 162(11):6392–400. 10352252

[72] Malipiero U Frei K Spanaus KS Agresti C Lassmann H Hahne M Myelin oligodendrocyte glycoprotein-induced autoimmune encephalomyelitis is chronic/relapsing in perforin knockout mice, but monophasic in Fas- and Fas ligand-deficient lpr and gld mice. Eur J Immunol (1997) 27(12):3151–60. 10.1002/eji.1830271211 9464800

[73] Sabelko KA Kelly KA Nahm MH Cross AH Russell JH. Fas and Fas ligand enhance the pathogenesis of experimental allergic encephalomyelitis, but are not essential for immune privilege in the central nervous system. J Immunol (1997) 159(7):3096–9. 9317103

[74] Waldner H Sobel RA Howard E Kuchroo VK. Fas- and FasL-deficient mice are resistant to induction of autoimmune encephalomyelitis. J Immunol (1997) 159(7):3100–3. 9317104

[75] Sabelko-Downes KA Cross AH Russell JH. Dual role for Fas ligand in the initiation of and recovery from experimental allergic encephalomyelitis. J Exp Med (1999) 189(8):1195–205. 10.1084/jem.189.8.1195 10209037 PMC2193027

[76] Zipp F Krammer PH Weller M Immune (dys)regulation in multiple sclerosis: role of the CD95-CD95 ligand system. Immunol Today (1999) 20(12):550–4. 10.1016/S0167-5699(99)01545-5 10562705

[77] Gomes AC Morris M Stawiarz L Jonsson G Putheti P Bronge L Decreased levels of CD95 and caspase-8 mRNA in multiple sclerosis patients with gadolinium-enhancing lesions on MRI. Neurosci Lett (2003) 352(2):101–4. 10.1016/j.neulet.2003.08.030 14625033

[78] Balashov KE Rottman JB Weiner HL Hancock WW. CCR5(+) and CXCR3(+) T cells are increased in multiple sclerosis and their ligands MIP-1alpha and IP-10 are expressed in demyelinating brain lesions. Proc Natl Acad Sci U S A (1999) 96(12):6873–8. 10.1073/pnas.96.12.6873 10359806 PMC22009

[79] Teleshova N Pashenkov M Huang YM Soderstrom M Kivisakk P Kostulas V Multiple sclerosis and optic neuritis: CCR5 and CXCR3 expressing T cells are augmented in blood and cerebrospinal fluid. J Neurol (2002) 249(6):723–9. 10.1007/s00415-002-0699-z 12111306

[80] Julia E Montalban X Al-Zayat H Issazadeh-Navikas S Goertsches R Martin R Deficient Fas expression by CD4+ CCR5+ T cells in multiple sclerosis. J Neuroimmunol (2006) 180(1–2):147–58. 10.1016/j.jneuroim.2006.07.001 16899302

[81] Sharief MK. Increased cellular expression of the caspase inhibitor FLIP in intrathecal lymphocytes from patients with multiple sclerosis. J Neuroimmunol (2000) 111(1–2):203–9. 10.1016/S0165-5728(00)00310-6 11063839

[82] Semra YK Seidi OA Sharief MK. Overexpression of the apoptosis inhibitor FLIP in T cells correlates with disease activity in multiple sclerosis. J Neuroimmunol (2001) 113(2):268–74. 10.1016/S0165-5728(00)00443-4 11164911

[83] Macchi B Matteucci C Nocentini U Caltagirone C Mastino A. Impaired apoptosis in mitogen-stimulated lymphocytes of patients with multiple sclerosis. Neuroreport (1999) 10(2):399–402. 10.1097/00001756-199902050-00034 10203342

[84] Comi C Leone M Bonissoni S DeFranco S Bottarel F Mezzatesta C Defective T cell fas function in patients with multiple sclerosis. Neurology (2000) 55(7):921–7. 10.1212/WNL.55.7.921 11061245

[85] Huang WX Huang MP Gomes MA Hillert J. Apoptosis mediators fasL and TRAIL are upregulated in peripheral blood mononuclear cells in MS. Neurology (2000) 55(7):928–34. 10.1212/WNL.55.7.928 11061246

[86] Macchi B Matteucci C Nocentini U Tacconi S Pagnini V Mastino A Defective Fas ligand production in lymphocytes from MS patients. Neuroreport (2001) 12(18):4113–6. 10.1097/00001756-200112210-00050 11742248

[87] Lucas M Zayas MD De Costa AF Solano F Chadli A Dinca L A study of promoter and intronic markers of ApoI/Fas gene and the interaction with Fas ligand in relapsing multiple sclerosis. Eur Neurol (2004) 52(1):12–7. 10.1159/000079253 15218339

[88] Kantarci OH Hebrink DD Achenbach SJ Atkinson EJ de Andrade M McMurray CT CD95 polymorphisms are associated with susceptibility to MS in women. A population-based study of CD95 and CD95L in MS. J Neuroimmunol (2004) 146(1–2):162–70. 10.1016/j.jneuroim.2003.10.002 14698859

[89] van Veen T Kalkers NF Crusius JB van Winsen L Barkhof F Jongen PJ The FAS-670 polymorphism influences susceptibility to multiple sclerosis. J Neuroimmunol (2002) 128(1–2):95–100. 10.1016/S0165-5728(02)00163-7 12098516

[90] Langrish CL Chen Y Blumenschein WM Mattson J Basham B Sedgwick JD IL-23 drives a pathogenic T cell population that induces autoimmune inflammation. J Exp Med (2005) 201(2):233–40. 10.1084/jem.20041257 15657292 PMC2212798

[91] Harrington LE Hatton RD Mangan PR Turner H Murphy TL Murphy KM Interleukin 17-producing CD4+ effector T cells develop via a lineage distinct from the T helper type 1 and 2 lineages. Nat Immunol (2005) 6(11):1123–32. 10.1038/ni1254 16200070

[92] Park H Li Z Yang XO Chang SH Nurieva R Wang YH A distinct lineage of CD4 T cells regulates tissue inflammation by producing interleukin 17. Nat Immunol (2005) 6(11):1133–41. 10.1038/ni1261 16200068 PMC1618871

[93] Sakaguchi S Yamaguchi T Nomura T Ono M. Regulatory T cells and immune tolerance. Cell (2008) 133(5):775–87. 10.1016/j.cell.2008.05.009 18510923

[94] Lock C Hermans G Pedotti R Brendolan A Schadt E Garren H Gene-microarray analysis of multiple sclerosis lesions yields new targets validated in autoimmune encephalomyelitis. Nat Med (2002) 8(5):500–8. 10.1038/nm0502-500 11984595

[95] Komiyama Y Nakae S Matsuki T Nambu A Ishigame H Kakuta S IL-17 plays an important role in the development of experimental autoimmune encephalomyelitis. J Immunol (2006) 177(1):566–73. 10.4049/jimmunol.177.1.566 16785554

[96] Viglietta V Baecher-Allan C Weiner HL Hafler DA. Loss of functional suppression by CD4+CD25+ regulatory T cells in patients with multiple sclerosis. J Exp Med (2004) 199(7):971–9. 10.1084/jem.20031579 15067033 PMC2211881

[97] Borsellino G Kleinewietfeld M Di Mitri D Sternjak A Diamantini A Giometto R Expression of ectonucleotidase CD39 by Foxp3+ Treg cells: hydrolysis of extracellular ATP and immune suppression. Blood (2007) 110(4):1225–32. 10.1182/blood-2006-12-064527 17449799

[98] Shi G Ramaswamy M Vistica BP Cox CA Tan C Wawrousek EF Unlike Th1, Th17 cells mediate sustained autoimmune inflammation and are highly resistant to restimulation-induced cell death. J Immunol (2009) 183(11):7547–56. 10.4049/jimmunol.0900519 19890052 PMC2958176

[99] Yu Y Iclozan C Yamazaki T Yang X Anasetti C Dong C Abundant c-Fas-associated death domain-like interleukin-1-converting enzyme inhibitory protein expression determines resistance of T helper 17 cells to activation-induced cell death. Blood (2009) 114(5):1026–8. 10.1182/blood-2009-03-210153 19429865 PMC2721783

[100] Cencioni MT Santini S Ruocco G Borsellino G De Bardi M Grasso MG FAS-ligand regulates differential activation-induced cell death of human T-helper 1 and 17 cells in healthy donors and multiple sclerosis patients. Cell Death Dis (2015) 6:e1785 10.1038/cddis.2015.164 26068793 PMC4669842

[101] Panitch HS Hirsch RL Haley AS Johnson KP. Exacerbations of multiple sclerosis in patients treated with gamma interferon. Lancet (1987) 1(8538):893–5. 10.1016/S0140-6736(87)92863-7 2882294

[102] Adams DO. Molecular interactions in macrophage activation. Immunol Today (1989) 10(2):33–5. 10.1016/0167-5699(89)90298-3 2665771

[103] Steinman L. A brief history of T(H)17, the first major revision in the T(H)1/T(H)2 hypothesis of T cell-mediated tissue damage. Nat Med (2007) 13(2):139–45. 10.1038/nm1551 17290272

[104] Durelli L Conti L Clerico M Boselli D Contessa G Ripellino P T-helper 17 cells expand in multiple sclerosis and are inhibited by interferon-beta. Ann Neurol (2009) 65(5):499–509. 10.1002/ana.21652 19475668

[105] Duhen R Glatigny S Arbelaez CA Blair TC Oukka M Bettelli E Cutting edge: the pathogenicity of IFN-gamma-producing Th17 cells is independent of T-bet. J Immunol (2013) 190(9):4478–82. 10.4049/jimmunol.1203172 23543757 PMC3633668

[106] Peroumal D Abimannan T Tagirasa R Parida JR Singh SK Padhan P Inherent low Erk and p38 activity reduce Fas Ligand expression and degranulation in T helper 17 cells leading to activation induced cell death resistance. Oncotarget (2016) 7(34):54339–59. 10.18632/oncotarget.10913 27486885 PMC5342346

[107] Annibali V Ristori G Angelini DF Serafini B Mechelli R Cannoni S CD161(high)CD8+T cells bear pathogenetic potential in multiple sclerosis. Brain (2011) 134(Pt 2):542–54. 10.1093/brain/awq354 21216829

[108] Dusseaux M Martin E Serriari N Peguillet I Premel V Louis D Human MAIT cells are xenobiotic-resistant, tissue-targeted, CD161hi IL-17-secreting T cells. Blood (2011) 117(4):1250–9. 10.1182/blood-2010-08-303339 21084709

[109] Turtle CJ Swanson HM Fujii N Estey EH Riddell SR. A distinct subset of self-renewing human memory CD8+ T cells survives cytotoxic chemotherapy. Immunity (2009) 31(5):834–44. 10.1016/j.immuni.2009.09.015 19879163 PMC2789980

[110] Yolcu ES Ash S Kaminitz A Sagiv Y Askenasy N Yarkoni S. Apoptosis as a mechanism of T-regulatory cell homeostasis and suppression. Immunol Cell Biol (2008) 86(8):650–8. 10.1038/icb.2008.62 18794907

[111] Bluestone JA Abbas AK. Natural versus adaptive regulatory T cells. Nat Rev Immunol (2003) 3(3):253–7. 10.1038/nri1032 12658273

[112] Weiss EM Schmidt A Vobis D Garbi N Lahl K Mayer CT Foxp3-mediated suppression of CD95L expression confers resistance to activation-induced cell death in regulatory T cells. J Immunol (2011) 187(4):1684–91. 10.4049/jimmunol.1002321 21746966

[113] Beyer M Schultze JL CD4+CD25highFOXP3+ regulatory T cells in peripheral blood are primarily of effector memory phenotype. J Clin Oncol (2007) 25(18):2628–30. author reply 30–2 10.1200/JCO.2006.08.0192 17577047

[114] Wing K Ekmark A Karlsson H Rudin A Suri-Payer E. Characterization of human CD25+ CD4+ T cells in thymus, cord and adult blood. Immunology (2002) 106(2):190–9. 10.1046/j.1365-2567.2002.01412.x 12047748 PMC1782718

[115] Fritzsching B Oberle N Pauly E Geffers R Buer J Poschl J Naive regulatory T cells: a novel subpopulation defined by resistance toward CD95L-mediated cell death. Blood (2006) 108(10):3371–8. 10.1182/blood-2006-02-005660 16868256

[116] Fritzsching B Oberle N Eberhardt N Quick S Haas J Wildemann B In contrast to effector T cells, CD4+CD25+FoxP3+ regulatory T cells are highly susceptible to CD95 ligand- but not to TCR-mediated cell death. J Immunol (2005) 175(1):32–6. 10.4049/jimmunol.175.1.32 15972628

[117] Banz A Pontoux C Papiernik M. Modulation of Fas-dependent apoptosis: a dynamic process controlling both the persistence and death of CD4 regulatory T cells and effector T cells. J Immunol (2002) 169(2):750–7. 10.4049/jimmunol.169.2.750 12097377

[118] Zheng SG Wang JH Gray JD Soucier H Horwitz DA. Natural and induced CD4+CD25+ cells educate CD4+CD25− cells to develop suppressive activity: the role of IL-2, TGF-beta, and IL-10. J Immunol (2004) 172(9):5213–21. 10.4049/jimmunol.172.9.5213 15100259

[119] Zheng Y Josefowicz SZ Kas A Chu TT Gavin MA Rudensky AY. Genome-wide analysis of Foxp3 target genes in developing and mature regulatory T cells. Nature (2007) 445(7130):936–40. 10.1038/nature05563 17237761

[120] Li Q Wang Y Wang Y Zhou Q Chen K Wang YM Distinct different sensitivity of Treg and Th17 cells to Fas-mediated apoptosis signaling in patients with acute coronary syndrome. Int J Clin Exp Pathol (2013) 6(2):297–307. 23330016 PMC3544239

